# Perspectives of illicit marijuana growers and traders on commercial legalisation of marijuana in South Africa: considerations for policy formulation

**DOI:** 10.1186/s13011-021-00391-w

**Published:** 2021-06-26

**Authors:** Emmanuel Manu, Mbuyiselo Douglas, Mohlomi Jafta Ntsaba, Elvis Enowbeyang Tarkang

**Affiliations:** 1 Department of Population and Behavioural Sciences, School of Public Health, University of Health and Allied Sciences, Hohoe, Ghana; 2 Department of Public Health, Walter Sisulu University, Nelson Mandela Drive, Mthatha, South Africa; 3 Department of Nursing, Walter Sisulu University, Nelson Mandela Drive, Mthatha, South Africa

**Keywords:** Eastern Cape Province, Inqguza Hill Local Municipality, Marijuana cultivation, Marijuana legalisation, South Africa

## Abstract

**Background:**

Over the years, there has been a clarion call for legalising marijuana cultivation and trading for commercial purposes in South Africa. Proponents of the call argue that the criminalisation of commercial marijuana cultivation and trading has failed to halt illicit marijuana cultivation and trading. However, the views of those who economically benefit from the illicit marijuana trade on its legalisation remain empirically unsolicited.

**Objective:**

This study aimed to solicit the views of illegal marijuana growers and traders from two selected communities in the Eastern Cape Province of South Africa regarding the commercial legalisation of marijuana cultivation and trading to inform policy on the debate.

**Methods:**

In-depth key informant interview approach was used to interview 18 purposively sampled participants that were selected through the snowball sampling technique. The data were analysed using the thematic content analysis approach.

**Results:**

Participants had both positive and negative perceptions of the possible legalisation of marijuana cultivation and trading. On the positive side, participants indicated freedom from police, the opportunity to grow marijuana on a larger scale, capital acquisition for commercial marijuana cultivation and trading, and regulation of marijuana prices through unionisation as some of the benefits they would derive from the commercial legalisation of marijuana cultivation and trading. On the negative side, loss of their source of livelihood, fall in the price of marijuana and perceived increase in school drop-out rates were the concerns raised.

**Conclusion:**

While participants relished improvement in their economic fortunes upon commercial legalisation of marijuana cultivation and trading, they were also apprehensive about this policy due to the perceived consequences it may have on their livelihoods and communities. We, therefore, recommend that future discussions of the commercial legalisation of marijuana cultivation and trading in South Africa should be done in consultation with illicit marijuana growers and traders to ensure that their interests are safeguarded by such a policy.

**Supplementary Information:**

The online version contains supplementary material available at 10.1186/s13011-021-00391-w.

## Background

Over the past decade, there has been a clarion call for commercial legalisation of marijuana in many countries, including South Africa [1–3]. The call received a major boost when countries such as Australia and some states in the United States of America (USA) legalised marijuana cultivation and usage on medical grounds [4].

Hence, the relaxation of regulations on marijuana cultivation and usage in some countries has sparked a global movement for decriminalising commercial marijuana cultivation, trading and usage. This is against the backdrop that Civil Society Organisations (CSOs), through advocacy and lobbying, often influence policy change in many countries [5]. Proponents of commercial marijuana legalisation argue that such a move will not only empower the economies of poor countries but could also moderate the use of other hard illicit drugs such as opiates [6, 7]. For instance, recreational and medicinal marijuana sales allowed the Colorado government to collect more than $135 million in taxation revenue in the year 2015 alone [8]. To ensure that positive outcomes occur alongside the considerable economic boost that recreational marijuana legalisation may present, the generated tax revenue could be used to fund substance abuse and regulation of marijuana use [9]. Recreational marijuana legalisation could also result in the employment of more workers who can grow and package marijuana for sale, and in a country where the unemployment rate is high among rural dwellers, this could be a good opportunity. It could also present job opportunities to those who can work in educational and health promotion initiatives to promote the safe use of the drug [9].

In Africa, Malawi is the latest country to legalise commercial cultivation and trading of marijuana, with a host of countries considering a similar move [10, 11]. According to Mumbere and colleagues [11], the global market for medical marijuana is currently estimated at $150 billion and could reach $272 billion in 2028; hence, African countries should consider tapping into the market for much-needed revenue generation and job creation. Taking into consideration the slow pace of industrialisation leading to an overreliance on natural resources for development on the continent [12–14] in the midst of increased population growth, there is the need for African governments to find alternative sources of income generation for themselves and their citizens of which recreational marijuana legalisation could be considered. The need for recreational marijuana legalisation as an alternative source of employment and income generation has become increasingly necessary because of increased rural-urban migration due to lack of economic opportunities in rural settings, putting enormous pressure on the few resources in urban centres [15, 16].

Commercial marijuana cultivation and trading are prohibited in South Africa. The drug is classified as a schedule 2 drug, which makes its cultivation, trading and usage prohibited per the Drugs and Drug Trafficking Act 140 of 1992 [17] and the Prevention and Treatment of Substance Abuse Act, No 70 of 2008 [18]. Engaging in marijuana cultivation and trading is, therefore, a criminal offence and attracts long prison sentences [18, 19]. These Acts require law enforcement agents to seize, arrest culprits and destroy marijuana plantations. However, the criminalisation of commercial marijuana activities such as its cultivation and trading has failed to stop the practice as undeterred citizens continue to indulge in it. This has led to the call for its legalisation in the country.

The call for marijuana legalisation received a major boost through a high court ruling that permitted private marijuana cultivation and usage [20, 21]. This is against the backdrop that a bill has been tabled in the South African parliament to consider legalising marijuana on commercial grounds. Proponents of comprehensive marijuana legalisation in South Africa, that is, its cultivation, trading and usage, posit that the crackdown on drugs has failed. This is because, despite the criminalisation of illegal drug activities, South Africa remains a major illicit drug producing, trafficking and usage hub on the African continent [22, 23]. For instance, in the year 2010, persons who were admitted into treatment centres for drug abuse for the first time in their lives increased from 67 to 75%, highlighting an increase in the rate of substance abuse and its attendant health consequences in the country [24]. This indicates that the war on drugs has not lived up to expectation; hence, the need for harm reduction approaches such as decriminalisation of marijuana use. In support of recreational marijuana decriminalisation, Khan [25] argues that no restriction can prevent people from using the drug, hence, the need to legalise its commercial and recreational activities. Moreover, favourable augments have been made in support of the medicinal and commercial value of marijuana, thus, the need for its legalisation [26, 27].

Despite marijuana being an illicit drug under current laws [17, 18], its cultivation and trading take place along the coastal belt of the Kwazulu-Natal and Eastern Cape provinces and the Limpopo and Mpumalanga provinces [28–30]. Despite the calls for commercial and recreational legalisation of marijuana in South Africa, empirical evidence is lacking on the perceptions of illegal marijuana growers on such a policy. Also, studies on the subject have either focused on the medical legalisation of marijuana or on the end-user [31–35], neglecting those who economically depend on its illegality for economic gain [28]. Since illegal marijuana cultivation and trading are so central to the economic survival of inhabitants of communities such as the Inqguza Hill Local Municipality (IHLM) in the Eastern Cape province [28, 36], it is pertinent to empirically solicit the views of such people in the commercial marijuana legalisation discourse of the country. We, therefore, followed the qualitative reporting guidelines of O’Brien et al. [37] to empirically ascertain the perspectives of selected illicit marijuana growers and traders in the IHLM of South Africa on the call for commercial legalisation of marijuana activities in South Africa.

## Methods

### Context

The was study was conducted in two communities in the IHLM of the Eastern Cape Province of South Africa, known for their involvement in illicit marijuana cultivation and trading. The municipality has a population of 278,481 and a population density of 234 people per square kilometre [38]. The communities were chosen for the study due to the involvement of inhabitants in illegal marijuana cultivation and trading. Therefore, they are likely to be affected by possible commercial legislation of marijuana cultivation and trading, hence, the need to solicit their views on the topic.

### Research approach

The key informant approach, an expert source of information [39], was employed to conduct this study in March 2016. The formal role of key informants means that they possess the kind of information needed by the researcher and have access to the information desired, and have absorbed the information meaningfully. Moreover, the informant should be willing to communicate their knowledge to the interviewer and cooperate as fully as possible [39]. Based on the above criteria, experienced individuals who have been involved in illicit marijuana cultivation and trading for 5 years and stood to be affected by a change in policy regarding illicit marijuana cultivation were considered to be ideal participants for the study. By gaining the trust of community members through the involvement of data collectors who hail from the communities and interacting with community members for a length of time, willing marijuana cultivators and traders availed themselves for an interview as they felt safe to divulge their illicit dealings to the research team.

### Population and sampling

The source population consisted of all illegal marijuana growers and sellers in the two selected communities. To be included in the study, a participant had to be older than 18 years and be involved in illegal marijuana cultivation or trading for at least 5 years. A total of 18 participants, comprising 12 marijuana growers-cum-sellers and eight marijuana sellers, were purposively selected and interviewed. There was an unequal representation of gender for the eighteen (18) participants. Twelve (12) participants were males, while six (6) were females. No participant was less than 30 years of age. None of the participants had tertiary education, and none was formally employed. Illicit marijuana cultivation and trading was their main occupation, and they had all been in the trade for not less than five (5) years. Sixteen (16) were Christians, and two (2) practised African Traditional Religion.

Participants were recruited through the snowball sampling technique. This was done by first identifying a contact in each of the communities who then helped to recruit known marijuana growers or traders. The process continued until data saturation was reached. This was when new codes and themes were not emanating from the study as the research process was iterative [40]. Snowball sampling was considered the ideal sampling technique for the study since it was difficult to gain access to the study population through random recruitment [41]. The researchers, therefore, needed to rely on the rapport established with individuals in the communities to persuade and recruit other marijuana growers to voluntarily participate in the study.

To confirm that participants illegally cultivated or traded in marijuana, the Principal Investigator and two research assistants visited marijuana plantations of self-acclaimed illicit marijuana growers and ensured that they were involved in illicit marijuana cultivation. Similarly, the marijuana sellers were also visited in their homes to ascertain that they had marijuana in stock for sale and were also known by at least one other participant to be involved in illegal marijuana trading. Although a bit apprehensive, participants allowed us access to their farms and homes due to the rapport, we had created with them after immersing ourselves in the communities for more than a month before the study, and therefore, we gained their trust.

### Interview guide

An in-depth key informants’ interview guide (Additional file 1), constructed in English and translated verbatim into the IsiXhosa language, was used to conduct the interviews. The guide was developed by three research team members (EM, MD and MJN) and translated into the IsiXhosa language by a certified language translator from the Eastern Cape Department of Education, South Africa. The guide was used to collect information on the socio-demographic characteristics of participants and their opinions about the commercial legalisation of marijuana cultivation and trading in South Africa. A pilot study was conducted in a community with similar characteristics to the two communities where the actual study was conducted, and the appropriateness of the interview questions in answering the research question was established [42]. The tool was then modified based on experiences from the pilot study.

### Key informants’ interviews

The data collection team comprised of one PhD researcher (EM), two research assistants trained in qualitative data collection methods and one project supervisor (MD). Two trained research assistants, who were postgraduate students at the Faculty of Health Sciences of the Walter Sisulu University and fluent in English and IsiXhosa languages, collected the data in March 2016, under the guidance of the Principal Investigator. The assistants were trained in the data collection instrument and the process of conducting face-to-face key informant interviews by EM, MJN and MD.

Data collection was preceded by community entry, where community members were addressed on the nature and purpose of the study during community tribal court gatherings. This was to secure the safety of the research team and to identify key informants. However, data collection did not immediately commence after community entry. The Principal Investigator (PI), together with the two data collectors, stayed in each community for up to a month and informally interacted with community members by attending social gatherings to gain community members’ trust due to the sensitivity of the topic. After gaining the trust of community members, key informants were identified and recruited, who then assisted in the recruitment of subsequent participants. Fifteen marijuana growers from the two communities were initially recruited, but three felt uncomfortable to participate in the study and subsequently withdrew. The remaining 12 marijuana growers comprising eight males and four females, together with six marijuana sellers comprising two males and four females, were then interviewed. Prior to the interview, participants were assigned alphabets as unique identifiers, starting from the letter A. Marijuana growers from the first community were assigned alphabets starting from GC1, while marijuana growers from the second community were assigned alphabets, beginning with GC2. Hence, the first marijuana grower interviewee in the first community was assigned a unique code, GC1 A, while the first marijuana grower interviewed in the second community was coded GC2 A. Similarly, marijuana sellers from the first community were coded SC1 while those from the second community were coded SC2. The coding principle used in identifying marijuana growers was followed in identifying marijuana sellers from both communities. The unique codes allowed for easy tracking and matching of transcripts with their sources during coding and data analysis. Each interview session lasted between an hour and an hour and a half. All the interviews took place at safe and secluded locations. This ensured that participants felt comfortable enough to participate in the study. The interviews were recorded with an Olympus voice recorder, with the permission of participants.

### Data analysis

Thematic content analysis approach was used to analyse the data. Content analysis is a close inspection of text(s) to understand themes or perspectives [43]. The inductive coding process was adopted in this analysis [44]. The interview transcripts were first translated from the IsiXhosa language to English by a professional translator from the Eastern Cape Department of Education, South Africa, with 10 years experience in translation. We (EM, MD and MJN) ensured constant interaction with the translator to ensure that the interviewees’ exact words were used for interpretation to ensure methodological reflexivity [45]. The transcripts were first labelled to keep track of their source, that is, the communities and the individual participants they originated from. The transcripts were then independently thoroughly read by the researchers (EM and MD) to gain a general sense of the information. Coding was then done by writing all the applicable codes in the margins of transcripts, paragraph by paragraph. At the end of the independent coding process, a meeting was held where we (EM, MD and MJN) reviewed discrepancies between the two coders and revised discrepancies through consensus using the most expressive words for each set of codes. Related codes were then grouped under various themes as they emerged from the data analysis process. When further coding was not possible, data saturation was deemed to have been reached [40]. The themes, with their relevant quotes, were then used to structure the presentation of the results. Letters (1 or 2) were placed in front of each supporting quote in order to trace the community where the attributed participant hailed from.

### Trustworthiness

Guba [46] posits that in order to achieve trustworthiness in a qualitative study, the issue of truth-value (also credibility), applicability (transferability), consistency (dependability) and neutrality (confirmability) need to be addressed during the research process. Hence, we strived to adhere to these principles throughout the research process. For instance, the credibility of the study findings was achieved by spending enough time in the communities prior to data collection to gain the trust of participants, thereby opening them up for interviews and elicit credible responses. The lengthy stay in the communities also allowed for on-field member checking by visiting participants to confirm the credibility of the translation of the transcripts as the research process is iterative. Peer debriefers, who are experienced in qualitative research, were consulted, and their inputs were then incorporated into the research process to make the findings more credible. Transferability was ensured through a detailed description of the study’s setting and methodology to make it possible for its findings to be compared to studies from similar settings and methodological approach, albeit not being the aim of the naturalistic inquiry [46].

Moreover, stepwise replication of study findings led to dependability of the study findings. This was achieved by independent analyses of the datasets by three authors (EM, MD and MJN), after which meetings were arranged to discuss and merge study findings. Consistent communication took place between the data analysts before meetings to cross-check developing insights and decide the appropriate steps. Lastly, we ensured confirmability by being transparent with our study participants by explaining to them why we formulated and presented our questions and study findings the way we did for their approval. We ensured that data existed in support of every interpretation we made through a confirmability audit by an independent qualitative researcher.

## Results

The key findings that emerged from the data were grouped under two broad headings; perceived benefits and harm of legalising commercial marijuana to illegal marijuana growers and traders. On the positive side, it was found that freedom to engage in marijuana cultivation and trading, capital acquisition to indulge in commercial marijuana cultivation and trading and the regulation of marijuana prices through unionisation were the benefits illegal marijuana growers desired to gain from possible commercial legalisation of marijuana cultivation and trading. However, fear of takeover of the marijuana business by already established farmers, competition from other small-scale farmers leading to loss of loyal customers and loss of a source of livelihood, perceived fall in the price of marijuana due to lack of bargaining power, and a potential increase in school drop-out rates among children to engage in marijuana activities were the costs that illegal marijuana growers envisaged from possible commercial legalisation of the drug. The two broad headings (perceived negative and positive implications), together with their sub-themes, were not listed in an order of significance but were randomly arranged, with only two supporting quotes for each sub-theme irrespective of the frequency of quotations for the sub-theme, in order to avoid over-elaboration.

### Perceived positive implications of commercial marijuana legalisation

Participants stated some benefits that legalising commercial marijuana cultivation and trading would present. These benefits include freedom to engage in commercial marijuana cultivation, capital acquisition for commercial marijuana cultivation and the formation of unions to regulate marijuana price in their favour.

#### Freedom from the police

Although participants were apprehensive about the prospect of legalising commercial marijuana cultivation and trading, the benefits they perceive they would receive from such a policy has led them to consider embracing the notion. A female marijuana grower who was used to travelling long distances to sell marijuana explained how legalising the marijuana trade could liberate her and allow her to conduct business freely without being afraid of the police. She explained:

*Sometimes I hide it [marijuana] in my luggage from here [Community 2] to Johannesburg, which is very risky. You [the transporter] are always scared when you see a policeman approaching your car, so if it [commercial marijuana cultivation] is legalised, we [illegal marijuana growers] won’t fear the police again and will also not be imprisoned for dealing in it [illegal marijuana business]* (Female marijuana seller, Community 2, 49 years old)*.*

*This business [illicit marijuana cultivation] is risky because you may never know when the police will come to your house and arrest you or go to destroy your plantation in the bush. So, when the [marijuana] trade is legalised, there will be no need to live in fear. At least you won’t panic when you see a police officer approaching you* (Male marijuana grower, Community 1, 42 years old)*.*

#### Opportunity to grow marijuana on a larger scale

A male illegal marijuana grower also explained how commercial legalisation of marijuana cultivation and trading could present him with the opportunity to grow marijuana on a larger scale. He said,

*We [illegal marijuana growers] can increase the size of our [marijuana] farms without the fear of the police if it [marijuana cultivation] is legalised. As it stands now, you [marijuana grower] don’t want to attract the attention of the police or informants to yourself by cultivating a large field, but if it [commercial marijuana cultivation] is legalised, we will be free to do so. Imagine planting [marijuana] as big as you can without the police coming to spray your farm [with weedicides], that will be a big relief (*Male marijuana grower, Community 1, 42 years old).

#### Ease of capital acquisition for commercial marijuana cultivation and trading

Again, while marijuana farmers were initially somewhat wary of possible commercial legalisation of marijuana cultivation and trading, they believed that should it be legalised, it will present them with the opportunity to acquire loans to increase productivity. A male marijuana grower, for instance, predicted how he would capitalise on such an opportunity to secure funding from financial institutions. He narrated,

*I am sure that the banks will help us [marijuana growers] with some loans to invest in our [marijuana] farms if marijuana cultivation is legalised. They [banks] know the quality of marijuana we produce here [in these communities], so they [banks] will not hesitate to help us. With a reliable source of funding, we can also compete with other [commercial] farmers* (Male marijuana grower, Community 2, 50 years old).

A male key informant also opined that farmers would benefit from the legalisation of marijuana cultivation as they would now be bold to write to government funding agencies for funding. He was of the view that as a deprived community, farmers from the area will be in a good position to secure funding from organisations such as the National Youth Development Agency (NYDA) to invest in marijuana farming. He narrated,

*Yes, we [small scale illegal marijuana growers] will be able to compete with [large scale] farmers in other places. We [illegal marijuana farmers] are aware that there is a youth fund from the government that helps people to grow their business, but because the marijuana business is illegal, we can’t go for it. So, if marijuana cultivation is legalised, we [illegal marijuana growers] will feel comfortable to go and ask for help* (Male marijuana grower, Community 2, 48 years old)*.*

#### Regulation of marijuana prices through unionisation

Furthermore, the possibility of forming farmers’ unions to regulate the prices of marijuana, just like what other professional organisations and businesses do, was also another prospect participants were expecting from marijuana legalisation. A male marijuana grower from Community 2 was of the opinion that the legalisation of marijuana cultivation will present them with the opportunity to organise themselves as marijuana growers and sellers and regulate the price of marijuana to their advantage. He said,

*Well, if they [government] should legalise it [commercial marijuana cultivation], we [illegal marijuana growers] will also be able to form a union just like the bread sellers and other businesses do to make sure that the price of marijuana also favours us, so let’s wait and see what happens in the future* (Male marijuana grower, Community 2, 44 years old).

Commenting on the same issue, a female key informant in community 1 was of the view that marijuana growers could seize the opportunity to come together and form an association that would champion their course and maximise profit from their trade. She explained:

*I think we [illegal marijuana growers] will be able to come together to determine how much we want to sell a quantity of marijuana for. As it is now, we [individual growers] just bargain for ourselves, but if it is legal, we [as an association] can determine our own price* (Female marijuana grower, Community 1, 38 years old)*.*

### Perceived negative implications of marijuana legalisation

The major perceived negative effects of potential commercial marijuana legalisation from the perspective of illegal growers include perceived loss of their source of livelihood, fear of fall in the price of marijuana and increased school drop-out rates among children.

#### The takeover of the marijuana business by White commercial farmers (loss of their source of livelihood)

With regard to the perceived loss of a source of livelihood, while the assumption is often that illegal marijuana growers and traders would welcome legalisation and regularisation of their trade, participants were wary of such an idea as they saw it as a threat to their livelihood. To them, the legalisation of commercial marijuana cultivation and trading could lead to a loss of their tradition of illegal marijuana cultivation to White commercial farmers because these farmers have the capital and resources to undertake large-scale marijuana cultivation, which cannot be matched by illegal marijuana farmers. A male key informant said:

*White farmers would come with implements like sprinkler irrigation machines. They [White farmers] will also employ people and buy farms, thereby appropriating large quantities of land as they have lots of money, and thus, put us [small-scale marijuana farmers] at a disadvantage* (Male marijuana grower, Community 1, 34 years old).

Moreover, participants felt that the commercial legalisation of marijuana would lead to competition from other small scale marijuana growers that would emerge all over the country, and thus, lead to loss of their loyal customers and source of livelihood. They posited that loyal customers who would naturally travel long distances to buy from them would end up buying from easily accessible and cheaper sources or might even grow it themselves, pushing them out of business and robbing them of their livelihood. A female grower retorted:

*It [legalisation of commercial marijuana cultivation] would not be right. We [the illegal marijuana growers] would lose our customers because no one would want to go through the difficulties of travelling long distances to come here [Inqguza Hill Municipality] and buy it [marijuana]. People who come here to buy it will grow it themselves, and we [the illegal marijuana growers] will lose our customers and our source of livelihoods* (Female marijuana grower, Community 1, 44 years old)*.*

#### Fall in the price of marijuana

Another reason why participants did not wish for commercial legalisation of marijuana was the perceived fall in the price of marijuana once it is legalised. A female grower explained that they depend on the illegality of the commodity to keep its price high, as they are able to bargain for better prices from their desperate buyers who travel long distances to their communities to buy marijuana illegally. Legalising the cultivation and trading of the plant would, therefore, weaken their bargaining power. She explained:

*Our [illegal marijuana growers and sellers] profits will be reduced. People risk and travel to this place just to buy it [marijuana] because this is the only place they know [that marijuana is grown illegally]. We [illegal marijuana growers] are, therefore, able to ask for high prices and make a lot of money, but if it [marijuana] is allowed to be cultivated everywhere, there will be no need for people to travel here. The [illegal marijuana] business won’t be profitable again* (Female marijuana grower, Community 1, 38 years old).

Supporting the assertion of the female grower, a male key informant from Community 2 further retorted:

*My brother, it will not help us [illegal marijuana growers]. As it is now, we [the illegal growers] are able to ask for high prices because customers are desperate for it [marijuana]. They [customers] know that this is the only place where they can get quality marijuana, so they pay whatever price we ask. … But if it [commercial marijuana cultivation] is legalised, it [marijuana] will be all over the place, and we [the illegal growers] cannot control its price again* (Male marijuana grower, Community 2, 50 years old).

#### Increased school drop-out rates

Another key reason that served as a barrier to commercial marijuana legalisation among participants was that the illegality of the marijuana business served as a deterrent to children who might have loved to engage in its cultivation or trading at the expense of their education. Hence, legalising marijuana cultivation would erode such deterrence and encourage children to drop out of school to grow or sell marijuana commercially. A female marijuana seller explained:

*I don’t think it [legalisation of commercial marijuana cultivation] will help us [the community]. Our children will not want to go to school again because that [commercial marijuana business] will be an easy way to make money. They [school children] will just go to the bush and grow marijuana since they are going to school for money in the first place* (Female marijuana seller, Community 2, 44 years old)*.*

School-going children were also likely to be enticed to drop out of school to grow and sell the drug, as some of them were already involved in the illegal cultivation and trading of marijuana, though not on a large scale as full-time marijuana growers in the communities. Hence, legalising commercial marijuana cultivation will just encourage such children to drop out of school. A female marijuana grower opined:

*Our boys [in the community] will not go to school again if it [commercial marijuana cultivation] is legalised. Already, they [school children] have their own farms that they work on after school, so if it [commercial cultivation] is legalised and they [school children] are free to grow it [marijuana], the classrooms will be empty, and that will affect the future of this community* (Female marijuana grower, Community 1, 40 years old).

## Discussion

We explored the views of illicit marijuana growers and traders in the Inqguza Hill Local Municipality of South Africa regarding the commercial legalisation of marijuana cultivation and trading. Two broad themes were established: perceived positive and negative implications of marijuana legalisation. Thus, some participants saw some opportunities that commercial marijuana legalisation could present to them. However, they were apprehensive about legalising marijuana as it could erode their economic fortunes.

With reference to the perceived benefits that commercial legalisation of marijuana cultivation and trading would present, participants believed they could benefit from the opportunity to legally cultivate and trade in marijuana without fear of arrest and prosecutions. Participants’ dream of freely trading in marijuana upon its legalisation is understandable. Due to the criminalisation of marijuana and its associated arrest of perpetrators, South African farmers find it difficult to legally circulate their earnings accrued from illicit marijuana business into the formal economy and are thus not able to leverage on marijuana earnings for capital acquisition to maximize production [47]. Hence, decriminalisation of the drug could open up economic opportunities for farmers, as evidenced in North America and Europe [48], as farmers may not be arrested and the source of their income not questioned by anti-money laundering government agencies and financial institutions [49]. Thus, decriminalising marijuana cultivation and trading could provide illicit marijuana cultivators and traders an opportunity to freely conduct their business. However, usually, there is a delay in the development of police training materials on harm reduction upon legalisation of illicit substances leading to delay in the implementation of such policies by the police, who then continue using the law enforcement approach to deter people [50]. Hence, after the legalisation of marijuana in South Africa, the police would need to be taught in their training schools to shun abstinence-oriented approaches for harm reduction strategies in order to encourage those who previously engaged in illicit marijuana cultivation and trading to feel liberated to access the economic benefits associated with legal marijuana cultivation and trading.

Also, legally cultivating and trading marijuana could unlock the economic potential of governments and individual farmers that grow the plant illegally. This has been the case in Colorado, where both government revenue and individual growers’ income shot up considerably [51]. A similar assertion has been made in Australia. Analysing the economic and social benefit and cost of legalising marijuana in Queensland, Australia, the Bluegreen Economics [52], a socio-economic consultancy firm, argued that legalisation of marijuana for commercial purposes could open up economic opportunities for individuals involved in the industry. In Africa, marijuana historically was a legal economic crop until the twentieth century when prohibition laws were introduced by colonial rulers, making its value not fully realised as its legal and commercial marketing were banned [48]. Hence, commercial legalisation of marijuana could lead to the realisation of its full economic potential by farmers by tapping into both local and international markets. Moreover, commercial legalisation of marijuana could provide products that are different from those that exist for income generation by increasing the legal crop choices farmers have for income generation [48]. As the South African government faces job creation and revenue mobilisation challenges, leading to a poor economic outlook for the country [53], legalising commercial marijuana cultivation and trading could, therefore, unlock economic potentials for both the central government and citizens. This is particularly important considering the fact that alternative development interventions for marijuana growers, which aim to provide an incentive for farmers to switch to legal crop production, have consistently failed [48]. To ensure that commercial marijuana legalisation economically empowers illicit marijuana growers such as those in the context of the current study, there will be the need to introduce high yielding cultivar of the plant to farmers. At the same time, agricultural extension services should be extended to commercial marijuana growers so that they can harness modern farming methods to improve productivity.

Moreover, by legalising commercial marijuana cultivation and trading, farmers and traders will be presented with the opportunity to acquire capital from both private and government financial institutions to engage in large scale commercial marijuana cultivation and trading. Mbedzi and Simatele [54] provide a list of lending opportunities as well as the procedures to follow to acquire capital by micro and medium enterprises in the Eastern Cape Province. However, due to the illegality of the marijuana business, farmers cannot access funds from such institutions, thereby limiting their chances of increasing productivity. Hence, participants’ inability to engage in commercial marijuana cultivation until its legalisation is not misplaced. In order to make funds available to potential marijuana farmers in this regard, a special financial package at a lower interest rate should be provided to farmers by the government through various lending institutions. Moreover, historical bottlenecks that hamper support for most smallholder farmers in South Africa, such as poor distribution and use of resources leading to few farmers benefiting from such schemes [55], would need to be addressed in order to make such financial packages accessible to them. These policies, if implemented, could open doors for poor rural farmers who economically depend on illegal marijuana cultivation and trading to maximise profit.

Furthermore, illegal marijuana growers and traders were of the belief that should marijuana cultivation and trading be legalised on commercial grounds, they stand a better chance to form a union that will help them to regulate marijuana prices to their advantage. The power of organised labour in setting price floors has been empirically established [40, 56]: hence, marijuana legalisation could indeed improve the economic fortunes of illicit marijuana growers. It has also been noted that there has been a significant fall in the collective bargaining power of unions over the years [57]. As such, marijuana growers may not benefit significantly from commercial marijuana legalisation should their proposed unions be fragmented, making them less formidable to deal with the challenges that might come their way. However, to ensure that such a union has the legal backing for mass political and economic action for the welfare of its members, union leaders have to ensure that the union is formally constituted and recognised under the Labour Relations Act 66 of 1995 [58].

While participants liked some advantages that possible commercial marijuana legalisation could present to them, for some reasons, they did not wish for such a policy to be implemented. The reasons for their rejection of commercial marijuana legalisation were potential takeover of the marijuana business by already established commercial farmers, loss of loyal customers as a result of competition from several small-scale farmers, a fall in the price of the commodity and a potential increase in drop-out rates among school children in the area. With illegal marijuana cultivation and trading being the primary source of livelihood for most families in the two communities, losing their trade to already established commercial farmers with huge financial backing after its legalisation was a key issue to participants. The lack of on-farm infrastructure is not only a problem faced by illegal farmers in these communities but one of the main impediments that affect small scale Black South African farmers in the agri-business industry. The lack of on-farm infrastructure prevents farmers from undertaking large scale commercial production, and hence, they are confined to subsistence farming, which is less profitable [59]. For instance, while irrigation is an essential component of commercial agriculture, it is a major problem for emerging black farmers in South Africa. This is because it is capital intensive to install irrigation equipment, a capital that most Black farmers cannot raise [60]. Due to this, illegal small scale marijuana growers might not be able to undertake commercial marijuana cultivation after its legalisation; should they fail to secure funding, they would eventually lose out to already established commercial farmers who might want to diversify into marijuana farming after its legalisation.

Considering the fact that participants were scared of its legalisation because of how it could possibly affect its pricing and dry up opportunities while at the same time, they hope that there could be more opportunities for themselves. Certain policies need to be put in place to ensure that the interests of the farmers are protected. Firstly, it should be a requirement for marijuana growers to get a certification or licence, which should be subject to renewable annually to ensure compliance with regulations regarding commercial marijuana cultivation, as is the case in the State of California [61]. However, the cost of certification should be moderate in order not to deter would-be growers from applying. Moreover, licensing regulations should be uniform across all provinces in the country to avoid inconsistencies. They should also ensure that standard practices of farmers are not outlawed in the process of legalisation so as not to serve as a disincentive to farmers to get a legal licence [61]. Bodwitch and colleagues [62] argue that a stringent certification process leads to excluding small growers and only favours privileged, wealthier operations, which is a concern expressed by participants in the current study. Disinterest to seek a licence may also lead to an active unregulated market as black-market growers might operate with impunity, leading to a shortage of labour for on-the-books farmers [62]. In understanding the extent to which marijuana businesses support their local communities, there is the need for legalisation not to disregard the concerns and opinions of illicit smallholder farmers in the discourse of regulations regarding commercial marijuana legalisation. This would help to ensure that both the concerns and aspirations of illicit smallholder growers are catered for by such a policy.

Aside from the lack of financial muscle to compete with established commercial farmers, participants feared a potential decrease in the price of marijuana due to its widespread cultivation and trading after its legalisation. Participants believe that the legalisation of marijuana cultivation and trading on commercial grounds could lead to the emergence of many small-scale and commercial marijuana farmers. This will inevitably result in an oversupply of marijuana, leading to a decrease in its value. Participants’ fear of its price devaluation is not economically far-fetched as the effect of oversupply of a commodity on price decrease is an established economic principle. Hence, should marijuana be overproduced as a result of its legalisation, it will lead to a fall in its price [63]. The government should therefore consider setting up an agency to buy marijuana directly from farmers and be responsible for its supply onto the market for price regulation and stabilisation purposes, just as it is done in the Cocoa industry in Ghana [64].

Another concern raised by participants was the likelihood of an increase in school drop-out rates among children who might be lured into the business as a result of its lucrativeness. This concern is not farfetched as the school drop-out rate in South Africa is presently high in rural areas, including the IHLM [65]. Moreover, should marijuana be legalised, there is the possibility of school children opting to cultivate and sell marijuana over education. Aside dropping out of school as a result of direct involvement in commercial marijuana activities, increased marijuana usage due to its availability and acceptance could also indirectly influence school dropout rate, since marijuana use has been found to retard cognitive development in children and could cause disinterest in academic activities [66]. However, the concern of underaged children engaging in legal marijuana-related activities and usage could be mitigated through a properly regulated system that would mediate who qualifies to work in the legal marijuana business. In the State of Colorado, for instance, laws have been enacted to limit marijuana use to people over 21 years of age after its recreational legalisation [67]. The same can be done in South Africa as regards who qualify to engage in legal marijuana cultivation, trading and usage after its legalisation. But issues of ineffective regulation would first need to be addressed as the proliferation of black-market growers could lead to child involvement in illicit marijuana activities [9]. Even though commercial marijuana legalisation will exclude children, there is still the need to intensify drug use prevention campaigns across the country in order to protect the rights of children as enshrined in the constitution to ensure that they do not end up being negatively impacted by the availability and accessibility of marijuana in their communities [68].

### Considerations for policy formulation

Legalising commercial marijuana cultivation and trading in South Africa could have a number of implications, both positive and negative, to the grower and the state, as evidenced in our findings. Hence, in order to safeguard both growers and the state’s interest in the legalisation process, certain policy considerations need to be made.

With reference to freedom from police enforcement upon legalisation, there will be the need for re-training of the police and other law enforcement agents to ensure that they re-orient their services to focus on harm reduction strategies regarding marijuana use, as was the case in Vietnam [69]. Law enforcement should only focus on adherence to licensing regulations to ensure that licensed marijuana growers and sellers abide by the ethics of their work and operate within the confines of their certificate. Non-compliance to licensing regulations should, thus, lead to revocation of one’s licence. Community policing, as is done in some rural counties of California [70], should be encouraged among licensed marijuana growers and sellers to ensure that activities of black-market traders and growers do not draw the attention of law enforcement to such communities.

Also, the introduction of high-quality cultivars of marijuana, as was the case of California [71], coupled with the technological empowerment of licensed marijuana farmers, could aid in the realisation of the large-scale marijuana cultivation and trading dreams of participants after its legalisation. Such policies would ensure that farmers are economically empowered through commercial marijuana legalisation. In addition, lessons from the challenges faced by small-scale commercial farmers in the country should be considered as they prevent farmers from maximising their profit margins to ensure that such mistakes are not repeated in commercial marijuana cultivation and trading.

With reference to ease of capital acquisition, historical bottlenecks that hamper small-scale farmers in securing capital for expansion of their farms, especially Black farmers, will need to be addressed by both the government and union leaders. Some of these challenges include access to land for commercial farming and very few farmers benefitting from government funding support programmes [55, 72]. Thus, issues of land acquisition and access to capital for would-be marijuana growers should be improved.

With the hope that commercial marijuana legalisation could lead to regulating marijuana prices through unionisation, certain considerations need to be made in order to achieve such a goal. First, a union would need to be duly constituted and registered per the tenets of the laws governing labour unions in the country in order to empower. Also, the general public would need to be sensitised not to stigmatise members of such a union, considering the fact that marijuana has been classified as an illicit substance for generations, and those involved in illicit drug activities are stigmatised [73]. Thus, registered marijuana growers and traders should be accorded the same level of respect as those engaged in other legitimate enterprises in the country to ensure that they are not criminally profiled.

While certain policy considerations need to be made in order for participants to realise the perceived positive implications commercial marijuana legalisation stand to present, similar considerations need to be made in order to address the perceived negative implications of such a policy for illicit small-scale farmers. To ensure that the marijuana business is not taken over by already established farmers, licit commercial marijuana growing licences should be awarded to communities where marijuana is historically grown and other economically disadvantaged settings for a period of time until they are equipped enough to compete with already established farmers. Moreover, small-scale farmers should be empowered through training on modern methods of marijuana farming, and they should be supplied with the needed on-farm infrastructure to boost their capacity as modern farming methods have been found to transform livelihoods in rural African communities [74]. Also, when the sector is opened up to the general public, the quantity of marijuana to be cultivated by already established commercial farmers need to be capped to ensure that they do not push small-scale farmers out of business. The government also needs to ensure that the market for the produce of small-scale farmers is secured in order to keep farmers in business, as this has been a challenge for most small-scale farmers in South Africa [75].

With respect to the fear of the fall in marijuana prices, its pricing and supply into the market need to be controlled by a state regulatory body, in liaison with farmer unions to ensure that there is uniformity in pricing nationwide as was done to agricultural products during the great depression [76]. Also, measures such as stricter control of black-market trading after legalisation should be enforced. This would ensure that prices set by the state and farmer unions do not fluctuate as a result of the proliferation of marijuana through the black market.

To curb school drop-out rates among school children, the age restriction for marijuana growers, sellers and users should be enforced as is done in Canada and New Zealand [77, 78]. Licences of marijuana growers and sellers who may use children on their farms and shops should be revoked or fined, or both. This is to protect the rights of children as enshrined in the constitution. There should also be continuous education and sensitisation on the negative implications of the abuse of marijuana, especially among children. Moreover, setting a higher retail price for marijuana could curb its consumption, especially among children. For instance, it has been found that a 10% decline in price is likely to lead to approximately a 3% increase in cannabis use [79]. Hence, ensuring that marijuana prices are not lowered could potentially curtail its use.

## Conclusion

While participants desired improvement in their economic fortunes after the commercial legalisation of marijuana cultivation and trading, they were also apprehensive about such a policy due to the perceived consequences it may have on their livelihood and community. We, therefore, recommend that future deliberations regarding the commercial legalisation of marijuana cultivation and trading in South Africa be done in consultation with illicit marijuana growers and traders to ensure that their interest is safeguarded by such a policy.

## Supplementary Information

**Additional file 1.** Interview guide for illicit marijuana growers, sellers and gatekeepers.

## Data Availability

The datasets used and/or analysed during the current study are available from the corresponding author on reasonable request.

## Abbreviations

CSOs: Civil Society Organisations
IHLM: Ingquza Hill Local Municipality
OPEC: Organisation of Petroleum Exporting Countries
PhD: Doctor of philosophy
USA: United States of America
