# Supplementary material for: Perspectives of illicit marijuana growers and traders on commercial legalisation of marijuana in South Africa: considerations for policy formulation
Source: Subst Abuse Treat Prev Policy. 2021 Jun 26;16:54. doi: 10.1186/s13011-021-00391-w (PMC8236131; doi:10.1186/s13011-021-00391-w)
Supplement: Supplementary file 1 — Additional file 1. Interview guide for illicit marijuana growers, sellers and gatekeepers. [file 13011_2021_391_MOESM1_ESM.docx]

## APPENDIX I: INTERVIEW GUIDE FOR ILLICIT MARIJUANA GROWERS, SELLERS AND GATEKEEPERS ON COMMERCIAL LEGALISATION OF MARIJUANA

## Topic: Perspectives of illicit marijuana traders and sellers on commercial legalisation of marijuana in South Africa: considerations for policy formulation

## Group Discussion Guidelines:

*Good day. We have come to you to discuss your p***erspectives on the debate regarding commercial legalisation of marijuana cultivation and trading in South Africa:** *We would like the discussion we are about to hold to be informal, so there is no need for us to call on you before you respond. If you don’t understand a question please let us know. We are here to ask questions, listen and also make sure that you have made your opinion known as clear as possible.*

*We do ask that you keep your identity and remarks anonymous by not mentioning your name or your community’s name during the interview. If you want to respond to a question, mention the unique code we have given you (e.g. I am ‘’GC1’). Whatever we discuss here should remain here. We hope you will feel free to speak openly and honestly.*

*As explained, we will be recording the discussion with a voice recorder, because we don’t want to miss any of your comments. No one outside of this room, except the translator, will have access to these voice recordings and they will be kept in a safe place for some time before being destroyed later.*

*Helping are my assistants……………………….. and …………………………*

*One will be asking the questions and the other shall be taking notes and also helping in any way they can.*

## Topic Generation

## Facilitator explains:

As I said earlier, the main reason for this meeting is to solicit your opinion on the possibility of commercial legalisation of marijuana cultivation and trading in South Africa.

**PART A: Biographical information**

Q1. Gender………………………………………………………

Q2. Can you please tell us your age according to your last birthday? …………………

Q3. Can you please tell us your highest education level? ……………

Q4. Who do you live with/who takes care of you?

Q5. How long have you lived in this community?

**PART B: perspectives on commercial marijuana legalisation**

Q6. As an illegal marijuana grower, what is your opinion on legalisation of marijuana cultivation on a commercial scale? (Probe for perceived benefits and threats to the grower).is village?

Q7. As an illegal marijuana seller, what is your opinion on legalisation of marijuana cultivation and trading on a commercial scale? (Probe for perceived benefits and threats to the seller).is village?

Q8. As a community leader, what is your opinion on legalisation of marijuana cultivation and trading on a commercial scale? (Probe for perceived benefits and threats to the grower, the seller the community and the nation).

**PART C: Closing**

If you have any comments or questions for clarification you may ask.

- Thank you very much for your time and contributions.
- Have a good day
